# Silicon nanocrystal-based photonic crystal slabs with broadband and efficient directional light emission

**DOI:** 10.1038/s41598-017-05973-y

**Published:** 2017-07-18

**Authors:** L. Ondič, M. Varga, I. Pelant, J. Valenta, A. Kromka, R. G. Elliman

**Affiliations:** 1 Institute of Physics, Academy of Sciences of the Czech Republic, v.v.i., Cukrovarnická 10, 162 00 Prague 6, Czech Republic; 2 Charles University, Faculty of Mathematics and Physics, Department of Chemical Physics and Optics, Ke Karlovu 3, 121 16, Praha 2, Prague, Czech Republic; 3 Research School of Physics and Engineering, The Australian National University, Canberra, ACT 2601 Australia

## Abstract

Light extraction from a thin planar layer can be increased by introducing a two-dimensional periodic pattern on its surface. This structure, the so-called photonic crystal (PhC) slab, then not only enhances the extraction efficiency of light but can direct the extracted emission into desired angles. Careful design of the structures is important in order to have a spectral overlap of the emission with extraction (leaky) modes. We show that by fabricating PhC slabs with optimized dimensions from silicon nanocrystals (SiNCs) active layers, the extraction efficiency of vertical light emission from SiNCs at a particular wavelength can be enhanced ∼ 11 times compared to that of uncorrugated SiNCs-rich layer. More importantly, increased light emission can be obtained in a broad spectral range and, simultaneously, the extracted light can stay confined within relatively narrow angle around the normal to the sample plane. We demonstrate experimentally and theoretically that the physical origin of the enhancement is such that light originating from SiNCs first couples to leaky modes of the PhCs and is then efficiently extracted into the surrounding.

## Introduction

Light-emitting silicon nanocrystals (SiNCs) are a material of great importance due to their biocompatibility as well as the compatibility with standard electronic and communication platforms^1^. In contrast to bulk silicon the few nanometer-sized SiNCs can exhibit efficient luminescence even at room temperature due to quantum confinement effects^2–6^. By embedding the SiNCs into a solid transparent matrix, potential candidates for solar cells^7, 8^ and light-emitting diodes (LEDs) emitting in the visible and near-infrared spectral range can be created. For example, Maier-Flaig *et al*. have demonstrated electroluminescent LED devices with external quantum efficiency up to 1.1% as well as low turn-on voltages for SiNCs emitting in the red spectral range^9^. Cheng *et al*.^10^ reported highly efficient red electroluminescence from a Si nanocrystal-organic LED with external quantum efficiency ≈8.6%. Promising results have also been achieved recently for electroluminescent SiNCs embedded in a SiC film^11^ and in electroluminescent capacitive structures^12^. The external quantum efficiency can be further increased by structuring surface of these devices. One way to realize this is to create a rough surface on the device^13^, in which case the enhanced extraction originates from light scattering on random surface features which leads to Lambertian far-field radiation pattern. For some applications, however, a directed or more confined output of light is desirable. This is typically realized by using sophisticated optical elements attached to the top of the LED. On the other hand, structuring the surface of these devices in a periodic manner offers the potential not only for further increase of the external quantum efficiency but also for controlling the radiation pattern of the extracted luminescence. Periodic structuring of the surface may thus enable both efficient light extraction and light’focusing/confining’ in one device.

Photonic crystal (PhC) slabs^14^, structures with two-dimensional (2D) periodicity and finite thickness, enable to increase light extraction efficiency from thin light emitting layers via the light-matter interaction^15–17^. If the light emission spectrally overlaps the leaky modes or the so-called guided resonances^18^ of the PhC, light can couple to these modes and be diffracted out of the structure at defined angles. In order to achieve this, the structure must be carefully designed considering all its physical properties, including the spatial distribution of the refractive index.

2D PhCs have been employed to enhance the performance of LEDs based on direct band-gap III-V materials and they were shown to compete with the rough-surface LEDs with respect to extraction efficiency^16, 19^. Various designs and the influence of the structural parameters were investigated experimentally and theoretically^17, 20, 21^. Periodic structures were also used to extract light emitted from bulk Si and SiNCs. Recently, it was shown that photonic crystal slabs on silicon with carefully chosen dimensions can lead to very high extracted photoluminescence (PL) intensity^22^. For the SiNCs, for example, around 4-fold extraction enhancement was achieved for vertical electroluminescence of Si nanoclusters having peak emission in the near IR region (900–1000 nm)^23^. Important for the realization of Si-compatible efficient light emitters at telecom wavelengths was PhC enhanced extraction of the emission line of Er embedded in SiNCs^24, 25^. We have also shown that PL of red/NIR light emitting SiNCs embedded in SiO_2_ matrix can be increased by structuring its surface into a 2D PhC with square lattice symmetry^26^. This resulted in an 8-fold enhancement in extraction for light outcoupled in the vertical direction, however, the extraction into other angles was rather low. Furthermore, due to the relatively low refractive index of the SiNCs-rich layer, the TE and TM leaky modes used as extraction channels overlapped spectrally and thus the extracted luminescence was spectrally narrow. In order to enhance the extraction efficiency within a broader spectral range, the TE and TM resonances must be spectrally separated further from each. As computer simulation suggests, one way to achieve this is to increase refractive index of the material.

Here we present results of enhanced light emission from SiNCs/SiO_2_-based PhC slabs with dimensions carefully designed to maximize the light extraction while keeping most of the active layer untouched. The PhCs were fabricated with high Si implantation dose in order to increase the refractive index value of the SiNC-rich SiO_2_ layer up to 1.8 and to obtain spectral separation of the TE and TM fundamental leaky modes. As a result, the emission extracted via leaky modes was spectrally broad and at the same time confined within a relatively narrow extraction cone around the normal to the sample plane. By comparing the experimental and computational results we show that the enhancement originates from the coupling of light emitted by SiNCs into the leaky modes of the PhCs which are efficiently extracted into surrounding. Our results can be employed to enhance light extraction from SiNCs-based LEDs.

## Samples

**Figure 1:**
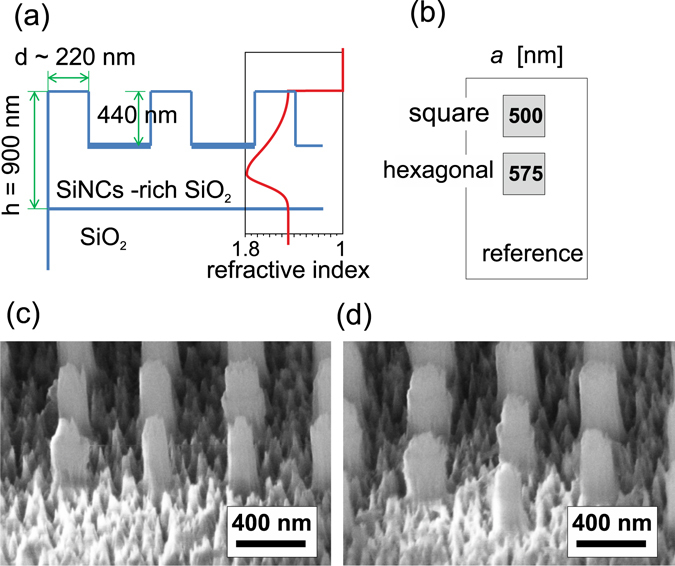
(**a**) Schematic cross-section of the PhC samples with their dimensions. (**b**) Layout of the sample consisting of square and hexagonal lattice PhCs with the lattice constant *a* (each having an area of 1 mm^2^) fabricated on the SiNCs-rich SiO_2_ layer surrounded by a 460 nm thick unpatterned reference SiNCs-rich layer which remained after the etching. (**c,d**) SEM images of the fabricated PhCs with square and hexagonal lattice symmetry.

The fabricated SiNCs-rich ≈900 nm thick layer was buried just beneath the silica substrate surface and possessed a bi-Gaussian distribution of SiNCs. The spatial distribution of SiNCs defines the spatial distribution of the refractive index which is schematically depicted in Fig. 1(a). The refractive index peaks at around 1.8 approximately 600 nm beneath the surface with standard deviations of the bi-Gaussian function being 186 nm (closer to the surface) and 137 nm.

PhC structures were etched into the surface of the SiNC-rich layer by the means of electron beam lithography (EBL) and subsequent reactive ion etching (RIE). The schematic cross-section and layout of the fabricated sample are shown in Fig. 1(a) and (b), respectively. Two PhC structures were prepared on the surface of the SiNC-rich layer, one possessing square lattice symmetry with lattice constant *a* = 500 nm (Fig. 1(c)) and the second one having hexagonal symmetry with *a* = 577 nm (Fig. 1(d)). The mean diameter of the columns is ≈220 nm (evaluated from SEM images) and their height is approximately 440 nm (evaluated from AFM measurements). As it is evident from the SEM images, the columns have relatively poor structural quality due to the long duration of the etching. Nevertheless, as demonstrated in the following section, even such an imperfect structure can possess high performance with respect to the light extraction.

PhC structures composed of columns ordered with a square and hexagonal symmetry were designed to achieve light extraction within a broad spectral range. Dimensions of the PhCs were optimized based on our previous experience^27, 28^ and based on the results of other authors^17, 20, 29^ as follows. The refractive index of the layer was chosen such that the TE and TM modes were spectrally well separated. Dimensions were chosen such that an overlap of both TE and TM fundamental leaky modes with the PL emission of the SiNCs was obtained. Due to the energy profile of the fundamental modes, which basically follows the spatial distribution of the NCs, coupling of the luminescence into these modes is very efficient. The final height of the PhC columns controlled by the etching time was chosen to maximize the extraction efficiency but also to keep intact as large a portion of the active layer with high density of SiNCs as possible. Even though almost half of the total thickness of the layer was removed (440 nm from 900 nm), the PhC contains more than 76% of the SiNCs from the original unetched sample thanks to the asymmetric SiNCs spatial distribution. Thus most of the SiNCs active layer remained also after etching of the sample and could feed the leaky modes. Dimensions of the PhCs were calculated using a rigorous coupled wave analysis technique (software DiffractMOD, RSoft) taking into account the parameters of the SiNCs-rich layer (refractive index, thickness, surrounding environment).

## Results and Discussion

**Figure 2:**
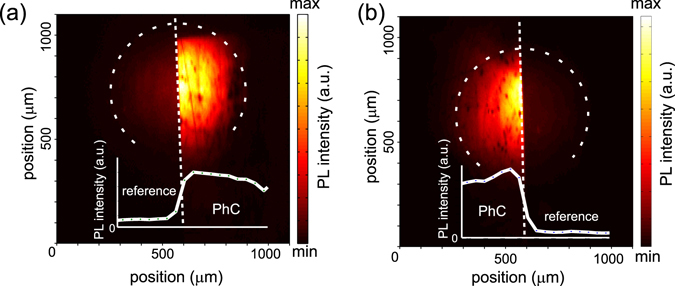
PL images at the interface (dotted line) of the unpatterned reference layer and (**a**) the square/(**b**) the hexagonal PhC structures. The PL images were obtained by exciting the samples with laser and detecting luminescence with an objective (NA = 0.13) coupled with a CCD camera, spectrally integrated within 500–1000 nm. Dashed circles depict the edges of the excitation beam with approximately Gaussian intensity profile. Insets show the PL at the vicinity of the boundary of the PhC structure and the planar part of the sample.

First, the qualitative performance of the samples regarding the light extraction was evaluated by measuring a direct PL image of the sample from the top. Figure 2 shows spectrally integrated PL emission of the SiNC-rich layer at the interface of the PhC layer and the surrounding etched uncorrugated (but slightly roughened) layer. The emission from the PhC part of the sample is greatly enhanced compared to the uncorrugated layer due to diffraction of the emission into air via the interaction with the PhC periodicity.

**Figure 3:**
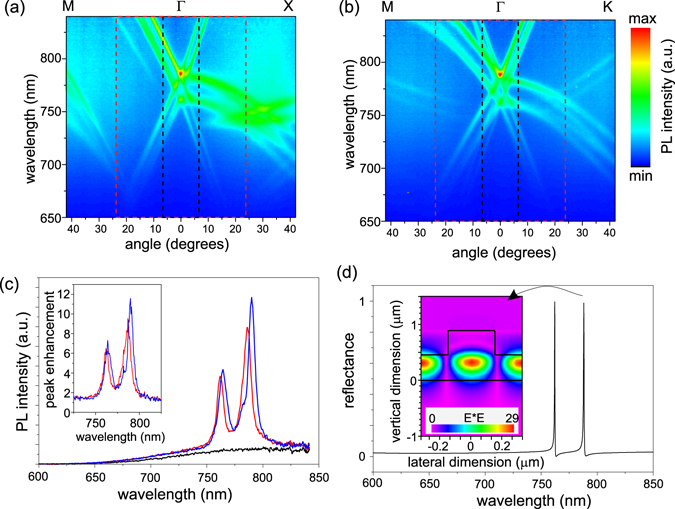
(**a,b**) Angle-resolved PL spectra of the studied PhC slabs having (**a**) square and (**b**) hexagonal lattice symmetry. The spectra were measured along the relevant high symmetry crystal directions. Angle of 0° is normal to the sample surface. The black and red rectangles plotted around the vertical (zero) detection angle depict the collection half-angles covered with the NA = 0.12 and NA = 0.4 objectives, respectively, used in the micro-PL setup. (**c**) Comparison of the vertically extracted PL emission spectra (detection angle of 0°) of the square (red) and hexagonal (blue) PhCs on the SiNCs-rich layer and the reference (black). Inset: Peak enhancement factor. (**d**) RCWA simulation of the normal incidence reflectance on the square lattice PhC showing the vertically extracted leaky mode resonances. Inset: Amplitude of the electric field of the TE fundamental mode in the square lattice PhC along the cut through the middle of computational cell.

Next, in order to map photonic band diagrams of the leaky modes, the fabricated PhC slabs were investigated by the means of angle-resolved PL spectroscopy. Figure 3(a) and (b) shows PL intensity as a function of the wavelength and an angle of extraction for the square and hexagonal lattice PhCs, respectively. Leaky modes of the photonic structures are revealed as the clearly visible intense PL bands superimposed on the typical spectrally broad PL spectrum of the SiNCs. Using this technique, where light emitted from the embedded light source serves as a probe of the leaky modes, only the modes spectrally overlapping the PL spectrum of the SiNCs are detected and thus the measured angle-resolved PL spectra basically reveal a part of the photonic band diagram of the studied sample. The results of the measurement confirm that the sample was well designed and that the PL emission spectrum of the SiNCs overlaps the leaky modes of the PhC thus securing an efficient light-matter interaction. These modes are guided modes in the case of the unpatterned reference layer but the formation of the PhC by surface patterning allows their out-coupling via diffraction.

Splitting into TE and TM modes is evidenced in the measured angle-resolved PL spectra (Fig. 3(a,b)) by the presence of two qualitatively similar radiation patterns which are spectrally shifted with respect to each other (TE being shifted towards longer wavelengths with respect to the TM modes). Compared with the PhCs with low peak value of refractive index studied in ref. 26, the PhCs studied here provide spectrally well separated channels for light of different wavelengths to escape from the layer and therefore they enable to extract light within a broader spectral range. The TE/TM splitting is clearly visible in the spectra of the vertically (zero angle) extracted PL shown in Fig. 3(c). Due to degeneracy of the modes at the Γ− point (modes propagating in vertical direction) originating from the band-folding of the waveguide modes^17, 27^ the highest extraction efficiency is in the direction normal to the sample plane. The highest peak enhancement factor of ≈ 11.8 (inset of Fig. 3(c)) was achieved for the TE leaky mode of the PhC with hexagonal symmetry. We define the peak enhancement factor (*F*_*Enh*_) as a ratio of the PL spectrum detected from the PhC with respect to the uncorrucated layer of SiNCs that remained after the etching. The uncorrugated etched layer will be taken as a reference for the evaluation of the performance of the PhC due to two main reasons: (i) roughness introduced during the etching is similar for both the PhC and the reference; (ii) the square and hexagonal PhCs (columns + the SiNCs-rich layer underneath) contain only 4.9% and 4.3% more SiNCs, respectively, than the etched reference layer (the 460 nm thick SiNCs-rich layer remaining after etching which surrounds the PhCs) as we have computed taking into account the spatial distribution of SiNCs. Due to this, only a small correction is needed to the measured data to extract the photonic effect of the periodic structure on the enhancement factor. Whereas, if the bulk unetched sample were considered as a reference, the situation would be that the bulk-sample contained 23.1% (23.6%) more SiNCs than the square (hexagonal) PhC and, more importantly, the different roughness would have to be considered too. Another effect that may increase the PL intensity of the PhC sample is resonant coupling of the incident excitation laser into the structure^30^. In the above presented measurements, the collimated excitation beam (325 nm) was incident normally on the samples. We have verified by computer simulation that for this experimental configuration the investigated PhC structures do not support leaky modes and thus the laser was not resonantly coupled into the PhCs. Furthermore, this effect occurs mainly when very low excitation power is employed. In our case, however, the PL of the SiNCs was almost saturated. An experimental evidence that the effect of the excitation laser resonant coupling along with other factors (e.g. increased surface of the PhC compared to the smooth layer) are negligible compared to the effect of the extraction via leaky modes is that the PL spectra are almost identical at other wavelengths. In summary, the extraction enhancement via leaky modes *F*_*Phc*_ can be extracted such that ${F}_{PhCHex}={F}_{Enh}/1.049$ for the hexagonal or ${F}_{PhCSq}={F}_{Enh}/1.043$ for the square lattice arising from a different amount of SiNCs in PhC samples and the reference. For the hexagonal lattice than ${F}_{PhCHex}=11.2$ and ${F}_{PhCSq}=9.2$. The enhancement factors are higher for the hexagonal lattice due to higher mode degeneracy at the Γ-point compared to that of the square lattice.

Figure 3(d) shows reflection spectra computed for the normal angle of incidence on the PhC with the square lattice symmetry that have a character of Fano resonances^18, 31, 32^ and identifies the leaky modes of the structure. The computed spectra show that the leaky mode resonances spectrally perfectly coincide with the measured ones shown in Fig. 3(c). The difference in the width of the resonances is due to the fact that the simulation does not include optical losses present in the real system and also does not account for the imperfect shape of the columns. The agreement of the simulation and measurement shows that the effect of the enhanced light extraction arises from the presence of the periodic structure and that it is not a result of some random scattering on imperfections or roughness. Furthermore, as expected from the design of the structure, simulation of the TE mode electric-field amplitude for the square lattice PhC (inset of Fig. 3(d)) confirmed that the extraction (leaky) modes are the fundamental modes with only one maximum in the intensity profile, consistent with the spatial distribution. The simulation performed for the hexagonal lattice PhC showed qualitatively similar results.

**Figure 4:**
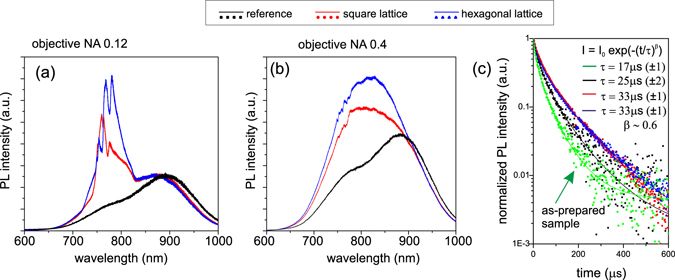
Comparison of the micro-PL spectra of the square and hexagonal PhCs on the SiNCs-rich layer and the reference measured within different collection angles. PL emission measured with (**a**) objective NA = 0.12 with the collection half angle of 6.9° and (**b**) objective NA = 0.4 with the collection half angle of 23.6°. (**c**) PL decay of the unpatterned sample (black symbols) compared to the PhC structures with square (red symbols) and hexagonal symmetry (blue symbols) measured around the spectral maximum of the TE modes. PL decay of the as-prepared 900 nm thick SiNCs-rich layer is also plotted (green symbols). Decay curves were fitted with a stretched exponential function. Results of the fit are summarized in the Inset.

The performance of the samples within a broader extraction cone was tested with a micro-PL setup. A cw laser tuned at 442 nm was focused on the samples through an objective and the PL was collected via the same objective. Figure 4(a,b) shows the PL emission spectra of the PhCs and of the unpatterned reference measured with two objectives differing in numerical aperture (NA). By increasing the numerical aperture, the collection angle increases and thus more and more leaky modes are collected, summing up into the final shape of the PL curve. In the spectra measured with the objective having NA = 0.12 and collection half angle of 6.9° (Fig. 4(a)), the leaky modes manifest themselves as still relatively narrow peaks superimposed on a broad emission spectrum from the SiNCs-rich layer. For the objective with NA = 0.4 and collection half angle of 23.6°, the situation is clearly different (Fig. 4(b)) and the spectra do not show any narrow peaks. The sign of the leaky modes presence is the significant change of their overall shape compared to the reference layer. As evident from the Fig. 3(b), the maximum of the PhC PL spectrum is blue-shifted and its overall intensity is higher than that of the unpatterned reference sample. Light extracted via leaky modes combines with radiative modes (modes not totally reflected on the air-sample interface) and forms the measured shape of the spectrum. The leaky modes are located at spectral positions where the intensity of the luminescence spectrum from SiNCs decreases and thus compensate this effect. It should be noted that because the excitation laser is focused on the sample through the objective, the light rays forming the laser beam are incident at different angles on the sample depending on the NA of the objective. Therefore, some of the light rays can be resonantly coupled into the leaky modes of the PhC. However, the experimental results show that this effect is again negligible compared to the PL extraction enhancement via the leaky modes. If the laser resonant coupling were efficient, the PL intensity of the PhC would have to be much higher than that of the reference within the whole emission range, which is not the case for the spectral region of 900–1000 nm.

To compare quantitatively the results for both objectives employed in the micro-PL measurements, a full spectral (or integrated) PL intensity enhancement factor was evaluated. It is defined as the ratio of integrated PL intensity from the PhC and the integrated PL intensity from the unpatterned layer. Full spectral enhancement factors corrected on the different amount of SiNCs in the PhC and the reference and thus representing extraction enhancement via leaky modes were computed (see Table 1) using similar definition as for the peak enhancement factors above. More than 60% and more than 40% increase in the integrated PL intensity was achieved for the hexagonal and square PhCs, respectively, compared to the unpatterned layer measured in the collection half-angle of 6.9° (NA = 0.12). For the collection half-angle of 23.6° (NA = 0.4), the enhancement of the integrated PL extraction is still relatively high, ≈ 57% for the hexagonal and ≈ 35% for the square lattice. A decrease in the area enhancement factor with increasing the collection angle follows from the nature of the PhC band diagram and the leaky modes themselves. With increasing **k**-vector of the leaky modes, generally the radiative constant of the modes characterizing losses into the surroundings decreases and thus also reduces the outcoupling efficiency. This is evident also from the measured angle-resolved PL spectra (Fig. 3(a,b)) where clearly the PL intensity of the modes extracted at higher angles is lower compared to those extracted at angles close to the vertical direction.

**Table 1:** Peak (for the fiber) and area (for the objectives) enhancement factors *F*_*PhC*_.

	fiber	objectives	
	TE mode	NA = 0.12	NA = 0.4
square PhC	9.2	1.44	1.35
hexagonal PhC	11.2	1.63	1.57

Finally, PL decay of the SiNCs embedded within the PhCs, within the SiNCs-rich reference layer, and within the as-prepared 900 nm thick SiNCs-rich layer was experimentally evaluated. The signal was collected within a small extraction cone (NA = 0.13) in order to maximize the effect of the vertically extracted modes. Figure 4(c) shows the measured decay curves spectrally integrated around the maximum of the TE mode. The decay curves were fitted with a stretched exponential law $I(t)={I}_{0}\exp (-{(t/\tau )}^{\beta })$, where *τ* is a decay constant and *β* is a dispersion coefficient showing deviation of the system from a single exponential decay. One of the physical explanations of this decay may be due to size distribution of the SiNCs. Because the PL lifetime of SiNCs depends among other things on their size, the measured PL lifetime distribution reflects the active SiNCs distribution. Then the stretched exponential PL decay from the nanocrystal ensembles may be simply explained as a result from mathematically summing a distribution of single-exponential decays with widely different lifetimes^33^. The numerical results of the fit are shown in the inset of Fig. 4(c). The *β* coefficient is within the experimental accuracy similar for all the fits and it is comparable to the results obtained also on differently prepared SiNCs^33–36^. This is expected as it is a characteristic of the studied ensemble of SiNCs and cannot be affected by changing macroscopic (with respect to the size of the NCs) geometry of the sample. PL lifetime characterized by the decay constant *τ*, on the other hand, depends on the type of the sample. The faster PL lifetime of the as-prepared sample compared to the partly etched planar layer, marked as a reference throughout this paper, can be attributed to the difference in the amount of non-radiative defects in the two samples. The excited carriers can be trapped on these defects before recombining radiatively which decreases the PL lifetime. We hypothesize that during the annealing the non-radiative centers are “pushed away” towards the surface of the sample. Therefore, part of the non-radiative defects is removed by etching the upper part of the sample, which increases the PL lifetime. For the both PhC samples, the PL lifetime is longer than in the unpatterned SiNCs-rich layers. Increase in the emission lifetime of light emitters in 2D and 3D PhCs^37^ have been observed and explained as an effect of a photonic band gap^38, 39^ which inhibits the emission into the slab modes. In our case, however, the principle of extraction is via leaky modes and the increase in the lifetime can be qualitatively explained as follows. (More rigorous theoretical explanation of this effect is out of the scope of this paper.) Leaky modes collected with the objective are propagating in the direction normal or close to the sample plane normal. One of the characteristics of these modes is that their group velocity is very low and they are often referred to as slow light^40, 41^. For modes at the Γ− point, the group velocity even approaches zero^42, 43^ and these modes form standing waves in the PhC (see the simulated electric field distribution of the TE mode at the Γ− point plotted as the inset of Fig. 3(d)). Therefore it seems natural that light coupled into these slow modes bounces back and forth in the plane of the PhC and simultaneously it leaks to air which causes the lengthening of the PL decay time.

## Conclusions

In conclusion, we have shown that light emission from SiNCs embedded in the SiO_2_ layer can be greatly enhanced when a 2D PhC with well-chosen dimensions is fabricated on its surface. The best results were obtained for the PhC with hexagonal symmetry in which more than 11-fold enhancement in the intensity of vertically emitted modes compared to the PL intensity of the uncorrugated layer was achieved. As shown by the measured photonic band diagram, light emission was not only increased but also directed into defined directions and confined within a relatively narrow angle around the sample normal. Within the detection cone with half-angle of 6.9° and 23.6°, an increase by 63% and 57% was measured for the PL extracted via the hexagonal PhC compared to that emitted from the uncorrugated layer. These results may be applied in silicon photonics when constructing LEDs based on SiNCs.

## Methods

### Fabrication method

SiNCs-rich layers were fabricated by Si ions implantation into polished silica substrates followed by thermal annealing at 1100° C (for details see ref. 26). The implant energy of 400 keV with the implant fluence 4 × 10^17^ Si/cm^2^ were used. The fabricated SiNCs have a diameter of 4–5 nm (detected by Raman scattering^44^) and possess red/NIR room-temperature photoluminescence. Values of the refractive index along with its spatial distribution were extracted from transmission measurement by carefully fitting the Fabry-Perot resonances in the transmission spectrum of the planar uncorrugated layer.

PhC structures were etched into the surface of the SiNC-rich layer by the means of electron beam lithography (EBL) and subsequent reactive ion etching (RIE). First, the surface of the sample was covered with an electron sensitive polymer (PMMA, 100 nm). Then, a periodic matrix with the dimensions based on theoretical calculations was created by an electron beam (eLiNe workstation; Raith, Dortmund, Germany). Afterwards, a 70 nm thick gold layer was evaporated and processed by the lift-off strategy to create a mask for the sample etching. The etching was performed by capacitively coupled plasma RIE (Phantom LT CCP-RIE System, Trion Technology) in pure SF_6_ plasma at the power of 300 W and working pressure of 150 mTorr for 5 minutes. After the plasma etching, the gold mask was removed in aqua regia.

### Optical characterization methods

For direct PL images of the sample, the sample was excited with a laser diode (405 nm) and the PL emission collected from the top by an objective with NA = 0.13 was then directly imaged on a back-illuminated deep-depletion nitrogen-cooled CCD. A similar setup was employed for PL lifetime measurements, however, in this case the collected PL signal was spectrally resolved by a monochromator and directed onto the Hamamatsu gated detector. Angle-resolved PL spectra were measured by exciting the sample with a parallel beam from a cw 325 nm laser through the substrate incident at a non-resonant angle. Then the extracted PL emission was collected from the front side of the sample where the PhC crystal structure was located with an optical fiber with collection half-angle of less than 0.5°, which was rotated above the sample along high-symmetry directions. The extraction performance of the fabricated samples as a function of the solid detection angles was investigated employing a micro-PL setup with two different objectives. Namely, the objectives with NA = 0.12 (collection half angle of ≈ 6.9°) and NA = 0.4 (collection half angle of ≈ 23.6°) were employed. The principle of the setup is such that a single objective is always used both for excitation with 442 nm laser of the sample and also for a collection of the PL. Depending on the NA of the objective, the excitation laser is incident on the sample under different angles. It is therefore possible that some of the light rays from the incident beam can be resonantly coupled into the structure. However, as discussed in the main text, this effect is negligible compared to the extraction via leaky modes. It should be noted that the laser beam can be partly diffracted on the PhC that operates also as a typical diffraction grating at the employed excitation wavelengths. At the employed laser wavelengths, diffraction into only the first order of diffraction occurs. Nevertheless, this effect causes only a negligible increase of the excited volume. Where appropriate, spectra were corrected for the spectral response of the detection system. All the measurements were performed at room temperature.
